# Detrimental Effects of ApoE ε4 on Blood–Brain Barrier Integrity and Their Potential Implications on the Pathogenesis of Alzheimer’s Disease

**DOI:** 10.3390/cells12212512

**Published:** 2023-10-24

**Authors:** Kevin Kirchner, Linda Garvert, Luise Kühn, Sarah Bonk, Hans Jörgen Grabe, Sandra Van der Auwera

**Affiliations:** 1 Department of Psychiatry and Psychotherapy, University Medicine Greifswald, 17475 Greifswald, Germany; 2 German Centre for Neurodegenerative Diseases (DZNE), Partner Site Rostock/Greifswald, 17475 Greifswald, Germany

**Keywords:** apolipoprotein E, amyloid beta, blood–brain barrier, cyclophilin A, low-density lipoprotein receptor-related protein1, matrix metalloproteinase 9, tissue inhibitor of metalloproteinase 3

## Abstract

Alzheimer’s disease (AD) is a progressive neurodegenerative disease representing the most common type of dementia in older adults. The major risk factors include increased age, genetic predisposition and socioeconomic factors. Among the genetic factors, the apolipoprotein E (ApoE) ε4 allele poses the greatest risk. Growing evidence suggests that cerebrovascular dysfunctions, including blood–brain barrier (BBB) leakage, are also linked to AD pathology. Within the scope of this paper, we, therefore, look upon the relationship between ApoE, BBB integrity and AD. In doing so, both brain-derived and peripheral ApoE will be considered. Despite the considerable evidence for the involvement of brain-derived ApoE ε4 in AD, information about the effect of peripheral ApoE ε4 on the central nervous system is scarce. However, a recent study demonstrated that peripheral ApoE ε4 might be sufficient to impair brain functions and aggravate amyloid-beta pathogenesis independent from brain-based ApoE ε4 expression. Building upon recent literature, we provide an insight into the latest research that has enhanced the understanding of how ApoE ε4, secreted either in the brain or the periphery, influences BBB integrity and consequently affects AD pathogenesis. Subsequently, we propose a pathway model based on current literature and discuss future research perspectives.

## 1. Introduction

Alzheimer’s disease (AD) is a progressive neurodegenerative disease representing the most common type of neurodegenerative disorders worldwide [1]. The healthcare expenses for treating Alzheimer’s disease in 2020 are estimated at USD 321 billion for the United States alone, and it is anticipated that these costs will surpass USD 1 trillion by 2050 as the population ages [2]. AD accounts for 60% to 80% of dementia cases [3], and with increasing life expectancy [4], a further rise in prevalence can be expected [1]. Hence, the development of effective preventive or therapeutic solutions is a high priority for healthcare systems worldwide [1]. Over the last decades, researchers sought to enhance our understanding of the pathological mechanisms behind AD, which has led to the development of diverse hypotheses and research approaches. Major risk factors that have been identified are increased age, genetic predisposition and socioeconomic factors [5]. Genetic analyses, including large genome-wide association studies [6], have identified the apolipoprotein E (ApoE) ε4 allele as the strongest known genetic risk factor for AD, making it a main focus in AD research. Notably, ApoE is synthesised and secreted not only in the brain but also in peripheral tissues, especially the liver [7]. The question of how both brain-derived and peripheral ApoE (bApoE and pApoE, respectively) might affect the central nervous system and the pathogenesis of AD remains a topic of debate. Briefly, established mechanisms include the accumulation of extracellular amyloid beta (Aβ) peptides or hyperphosphorylated tau protein fibrillary aggregation (tangles) in the brain [8,9]. In addition, other mechanisms via vascular processes have been proposed [7,10,11,12,13,14].

Recent evidence from animal and human studies suggests that cerebrovascular dysfunctions, leading to blood–brain barrier (BBB) leakage, might also be associated with ApoE ε4 and AD pathology [13,14,15]. For instance, cerebral microinfarcts and microbleeds, which are associated with the disruption of structural connections in the brain [16], represent a common pathological feature in patients with dementia and cerebrovascular diseases [17]. The association between cerebrovascular dysfunctions and AD pathology is further supported by the joint risk factor profile of AD and cardiovascular diseases consisting of the ApoE ε4 genotype, diabetes, hypoperfusion, hypertension and hypercholesterolemia [15].

In this work, we intend to illuminate the mechanisms underlying the interplay between ApoE ε4, blood–brain barrier leakage and AD. To achieve this, we provide an overview of the hypothesised pathways and mechanisms illustrating how ApoE ε4, secreted either in the brain or peripheral tissues, might impact BBB integrity and, thus, affect AD pathogenesis. Subsequently, we propose a combined pathway model based on current literature and discuss future research perspectives.

## 2. Blood–Brain Barrier and ApoE—Structure and Function

### 2.1. Blood–Brain Barrier

**Figure 1:**
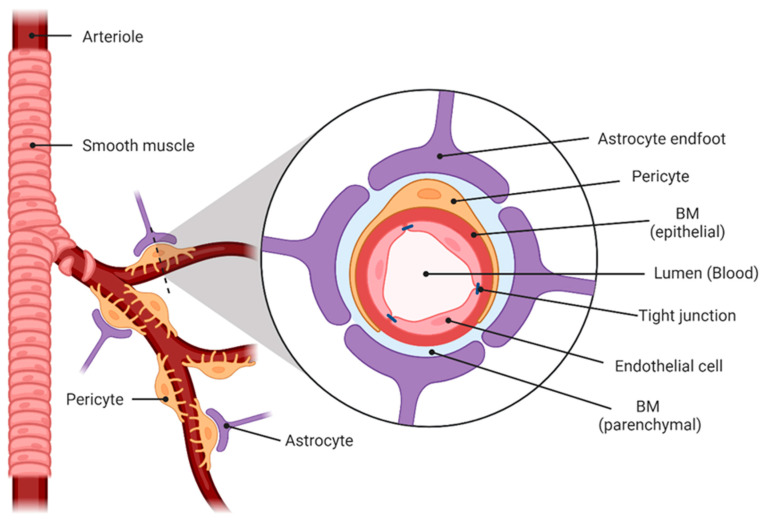
Visualization of the blood–brain barrier constituted within the microvasculature. BM = basement membrane. Created with BioRender.com accessed on 1 September 2023.

The blood–brain barrier is a highly selective semipermeable barrier primarily constituted within the microvasculature (capillaries), thereby separating the bloodstream from the brain and its extracellular fluid. It enables the controlled entry of nutrients into the brain and the removal of waste substances out of the brain, such as excessive glutamate and Aβ [7,18]. Unless a specific transport system is present, larger molecules, such as peptides and proteins, are unable to cross an intact blood–brain barrier [7]. On the cellular level, the BBB comprises predominantly a single layer of endothelial cells, surrounded by a basement membrane (BM) as well as pericytes and astrocytes [7,16,18] forming the neurovascular unit [19] (Figure 1). The endothelial cells are connected by tight junctions (TJs), specialised structures of various transmembrane proteins that form the semipermeable barrier crucial for preventing the free passage of substances [7,16]. Within the BBB, there are two distinct types of basement membranes (endothelial and parenchymal) that enclose the pericytes. Both types represent a specialised extracellular matrix that supports different functions such as structural support, cell anchoring and signalling transduction [20]. Pericytes are embedded in the BMs, where they regulate BBB permeability and the maintenance of TJs. Thus, they significantly impact cerebrovascular integrity and stability [7,16]. Astrocytes completely surround the capillaries with their endfeet, serve as the outer surface of the BBB and significantly contribute to the maintenance of the BBB [16]. They play a crucial role in neurotransmitter recycling, maintenance of tissue ion homeostasis and the regulation of synaptic transmission via the release of gliotransmitters [21].

### 2.2. Apolipoprotein E (ApoE)

ApoE was discovered in the early 1970s as a protein associated with cholesterol- and triglyceride-rich plasma proteins [7]. It is synthesised and secreted in the brain and peripheral tissues, predominantly the liver [7,22], and regulates lipid-related events [23]. The protein comprises 299 amino acids and exists in three major isoforms that differ only in one or two amino acids at positions 112 and 158: ApoE ε2 (cys112, cys158), ApoE ε3 (cys112, arg158) and ApoE ε4 (arg112, arg158). These variations modify both the structure and function of ApoE [24]. The ApoE ε4 allele has been identified as the strongest genetic risk factor for AD [9,25]. Carriers of the ApoE ε4 allele show an increased risk and decreased age of onset for AD as well as an earlier and ampler amyloid beta (Aβ) pathology compared with ApoE ε2 and ApoE ε3 allele carriers [23]. Amyloid beta denotes peptides derived from the amyloid-precursor protein (APP) and form the amyloid oligomers and plaques commonly seen in AD patients [3]. Although the function of Aβ is not well understood, the idea that Aβ plaque depositions are the main drivers for the development of AD already emerged in the 1980s with the amyloid cascade hypothesis by Hardy and Higgins, which subsequently dominated the research field during the past decades [26]. Aβ oligomers, in particular, were found to be associated with cognitive deficits [3], accelerated memory impairments and early cognitive decline in ApoE ε4 carriers [27] and were identified as primary drivers for neurotoxic processes in AD [28]. Of the three ApoE isoforms, ApoE ε4 shows the lowest Aβ clearance rate [7,29], which might be due to its weakened interaction with the low-density lipoprotein receptor (LDLR) and the LDLR-related protein 1 (LRP1), the two main contributors of ApoE-driven Aβ removal at the BBB [29]. Apart from its involvement in Aβ accumulation and clearance, studies have shown that bApoE ε4 is also associated with reduced BBB repair after traumatic brain injury, reduced TJ proteins in vitro, reduced pericyte coverage of blood vessels in human post-mortem tissue and increased BBB leakage [18].

As ApoE ε4 is secreted and synthesised not only in the brain but also in peripheral tissues such as the liver [22], it is debated whether pApoE ε4 might also affect the central nervous system and contribute to BBB degradation [23]. Although previous research generally assumed that bApoE ε4 and pApoE ε4 act independently and are separated by the BBB [18,30], a recent study by Liu and colleagues [23] demonstrated that pApoE ε4 might be sufficient to impair brain functions and aggravate Aβ pathogenesis. However, the exact roles of bApoE ε4 and pApoE ε4 and the BBB in the pathogenesis of AD still lack clear understanding.

## 3. The Impact of ApoE on the Blood–Brain Barrier

**Figure 2:**
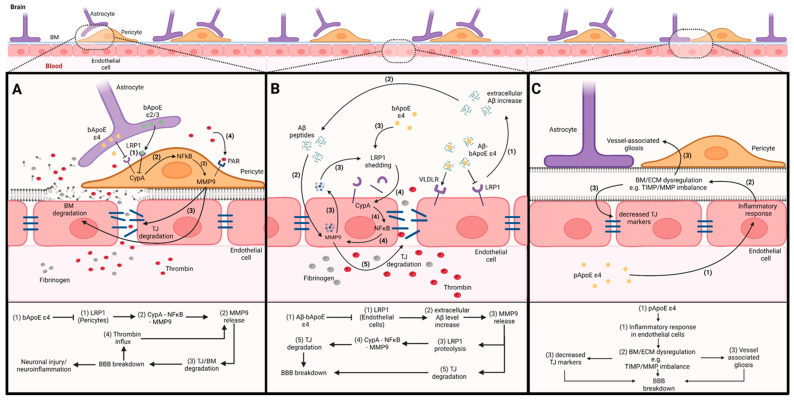
**Effect of ApoE ε4 on the BBB via three different mechanisms.** (**A**) **(bApoE ε4 via pericytes):** In contrast to bApoE ε2 and ε3, (1) bApoE ε4 is not able to bind to LRP1, which activates a CypA–NFκB–MMP9 pathway in pericytes and leads (2) to a release of MMP9 into the extracellular space. Here, MMP9 enzymatically degrades components of the ECM, which leads to (3) a BM/TJ breakdown resulting in an influx of neurotoxic proteins such as the coagulation factor thrombin. (4) Elevated levels of thrombin, in turn, might contribute to a further BBB leakage through MMP9 production via protease-activated receptors (PARs). (**B**) **(bApoE ε4 via endothelial cells):** (1) Aβ–bApoE ε4 compounds only interact weakly with LRP1 and are redirected to VLDLR, which internalizes Aβ–bApoE ε4 compounds at a reduced pace leading to increased Aβ levels (2). The exposure of endothelial cells to Aβ (3) promotes the release of MMP9 into the extracellular media. Subsequently, MMP9 impairs TJ maintenance via two different pathways. On the one hand, as bApoE ε4 (3) is less effective in the prevention of LRP1 shedding, MMP9 is able to proteolyse LRP1, which, in turn, promotes (4) the activation of the CypA–NFκB–MMP9 pathway in endothelial cells. On the other hand, elevated Aβ levels (2) lead to an elevated MMP9 release resulting in BM/TJ breakdown (5). (**C**) **(pApoE ε4):** (1) pAPOE ε4 may influence blood plasma factors, which contribute to inflammatory responses in endothelial cells. The resulting BM/ECM dysregulation (2), e.g., via TIMP/MMP imbalance, leads to (3) decreased TJ markers as well as (3) vessel-associated gliosis and promotes BBB breakdown. **Abbreviations:** Aβ = amyloid beta; BBB = blood–brain barrier; BM = basement membrane; bApoE = brain ApoE; CypA = cyclophilin A; ECM = extracellular matrix; LRP1 = low-density lipoprotein receptor-related protein 1; MMP9 = metalloproteinase 9; NFκB = nuclear factor-κB; pApoE = peripheral ApoE; PAR = protease-activated receptor; TIMP = tissue inhibitors of metalloproteinases; TJ = tight junction; VLDLR = very low density lipoprotein receptor. Created with BioRender.com accessed on 1 September 2023.

Dysfunctions of the BBB are linked to both the natural ageing process and several neurodegenerative disorders [23]. Studies indicate that both brain as well as peripheral ApoE ε4 might be able to induce neurodegenerative processes by affecting BBB integrity [7,11,12,18,23] and Aβ clearance [29,31,32]. To enhance our understanding of how ApoE ε4 might impact AD development, it is crucial to first elucidate the connections among AD, Aβ, ApoE and the BBB, along with their potential interactions. In the following, we will describe the postulated effects of ApoE ε4 on BBB integrity via three mechanisms: first, the impact of bApoE via pericytes (Figure 2A); second, the impact of bApoE via endothelial cells (Figure 2B); and third, the impact of pApoE (Figure 2C).

### 3.1. The Impact of bApoE on BBB Integrity via Pericytes

The following mechanisms are illustrated in Figure 2A. Studies have shown that the low binding affinity between human bApoE ε4 and LRP1, in contrast to bApoE ε2 and bApoE ε3, increases the intracellular CypA level in pericytes. This, in turn, leads to NFκB activation and finally to a release of MMP9 into the extracellular space (CypA–NFκB–MMP9 pathway) [7,11]. MMP9 belongs to a family of zinc-dependent extracellular matrix-remodelling endopeptidases and can degrade the capillary basement membrane as well as tight junction proteins (e.g., ZO-1, occludin, claudin-5) of the BBB [11]. Thus, excessive extracellular MMP9 can promote a BBB breakdown that allows the influx of blood-derived neurotoxic proteins, including the coagulation factor thrombin, into the brain [7,11]. Studies in vitro demonstrate that pericytes are thrombin-sensitive, MMP9-releasing cell types [33]. Thrombin can bind to pericytic protease-activated receptors (PARs), which triggers a process resulting in the additional release of MMP9. Hence, the influx of thrombin via an already damaged BBB might further promote BBB disruption due to elevated MMP9 release, ultimately favouring the development of a dysfunctional cycle [33].

In addition to these ApoE ε4-driven processes, pericytes might also play an active role in the removal of Aβ at the BBB through LRP1-mediated clearance (not depicted in the figure) [32]. However, studies reported a degeneration and loss of pericytes, particularly among subjects with AD. This decline seems to be a contributing factor in the progression of AD. Findings in mice suggest that the pericyte loss might result from an intracellular accumulation of Aβ as researchers observed decreased Aβ reuptake and diminished loss of pericytes after the inhibition of pericyte LRP1 [32]. Consequently, LRP1 might mediate both Aβ internalisation and pericyte cell death [32].

### 3.2. The Impact of bApoE on BBB Integrity via Endothelial Cells

For an illustration, see Figure 2B. In the endothelium, LRP1 regulates the transendothelial clearance of several neurotoxins, including Aβ. As prior studies have shown, compared with bApoE ε2 and ε3, the binding of Aβ to bApoE ε4 redirects the rapid clearance of Aβ from LRP1 to the very low density lipoprotein receptor (VLDLR), which internalises the Aβ–ApoE ε4 complexes at a reduced pace, resulting in elevated Aβ levels in the extracellular space [7,11]. Aβ accumulation, in turn, has been suggested to cause blood–brain barrier dysfunction by influencing multiple properties of endothelial cells [34]. For instance, Shackleton and colleagues postulate that the exposure of brain endothelial cells to Aβ triggers the release of MMP9 into the extracellular media [29]. Besides its role in breaking down and restructuring the extracellular matrix, MMP9 was also identified as a ligand for the transmembrane endothelial LRP1 and is able to proteolyse it [31,32]. As demonstrated in a prior study, treatment with recombinant MMP9 leads to a dose-dependent increase in proteolytic LDLR and LRP1 receptor shedding in endothelial cells. As studies indicate, bApoE ε4 is less efficient at preventing this proteolytic shedding compared with the other two isoforms [29]. Findings in mice showed that the inactivation and shedding of LRP1 can lead to a disintegration of the BBB via the activation of the self-autonomous CypA–NFκB–MMP9 pathway, which leads to a loss of important endothelial TJ proteins (ZO-1, occludin, claudin-5) and collagen IV [31]. Consequently, the disruption of the BBB caused by the reduction in brain endothelial LRP1 might initiate environmental milieu-altering processes that are associated with the influx of blood-derived neurotoxic proteins such as thrombin, albumin or fibrinogen and might result in a loss of neurons and the development of neuronal deficits [31]. Increased fibrinogen levels, in particular, have been suggested to be involved in this process, as studies found that reducing fibrinogen levels can considerably reverse neurodegenerative signatures in mice [23,35] (Figure 2B).

### 3.3. The Impact of pApoE on BBB Integrity

The following mechanisms are illustrated in Figure 2C. In contrast to bApoE, information about the impact of pApoE on the BBB is scarce. Previous research assumed that bApoE and pApoE act independently and are separated by the BBB. However, a recent and innovative study by Liu and colleagues showed that pApoE ε4 is sufficient to impair brain functions and to aggravate Aβ pathogenesis by using a conditional mouse model that expressed human ApoE ε3 or ε4 in the liver while lacking brain ApoE [23]. As the authors demonstrated, pApoE ε4 mice had a significantly compromised synaptic plasticity and cognition, increased BBB leakage, cerebrovascular impairments and increased vessel-associated gliosis compared with the pApoE ε3 mice. Via transcriptomic profiling and single-cell RNA sequencing analyses, they also found that the expression of pApoE ε4 impacted the extracellular matrix and BM, resulting in endothelial dysfunctions [23]. Additionally, pApoE ε4 exhibited a detrimental effect on immune responses within the gliovascular unit (extended neurovascular unit supplemented by oligodendrocytes and myelinated axons [19]) and was associated with a downregulation of genes crucial for endothelial cell function as well as maintaining the integrity of the BBB and the extracellular matrix [23]. Furthermore, their plasma proteome profiling revealed that immune-related and stress-related pathways were upregulated in pApoE ε4 mice, while anchoring junction, mitochondrial function and extracellular matrix pathways were downregulated in the brain endothelial cells of mice [23]. Notably, Liu et al. [23] observed that intravenously administering ApoE ε3 young plasma can improve cognitive function and reduce vessel-associated gliosis of aged wild-type mice, whereas exposure to pApoE ε4 young plasma affected BBB integrity without declining cognition. Additionally, they identified an upregulation of the tissue inhibitor of metalloproteinase 3 (TIMP3), an extracellular matrix-binding protein known to modulate the turnover of the extracellular matrix by inhibiting the activity of MMPs, in the plasma of ApoE ε3 mice [36]. They further observed that intravenously administering exogenous TIMP3 led to enhanced TJs in the presence of pApoE ε4 and concluded that ApoE ε4 plasma might have an impaired ability to maintain the integrity of the endothelial barrier via affecting the TIMP3-mediated pathway and/or the organisation of the extracellular matrix in comparison with pApoE ε3 [23]. 

Previous studies have already shown that the balance between MMPs and TIMPs plays a key role in the stability and normal function of the extracellular matrix [36], whereas imbalances were associated with vessel wall impairments [37]. Moreover, TIMP3 directly binds to LRP1, which mediates its endocytosis and degradation, as well as to a soluble form of LRP1 (sLRP1) [36], which is generated through the proteolytic shedding of LRP1 by MMP9 at the cell surface [29]. Although sLRP1 is still capable of binding ligands, it no longer possesses the ability to internalise or transport ligands, such as TIMP3, intracellularly [29], leading to elevated extracellular TIMP3 levels [36]. As an inhibitory effect of TIMP3 on MMP9 activity has been demonstrated [37], the impairment of the TIMP3-mediated pathway by pApoE ε4 and TIMP3 binding to sLRP1 might hinder the inhibition of MMP9. This, in turn, promotes the degradation of tight junctions and results in damage to the BBB. Although this is in line with observations of increased TIMP3 protein levels in the brains of AD patients and mouse models of AD [37,38], also contrary results have been reported [39]. As TIMP3 interacts with multiple other substrates in addition to MMP9 with reports indicating that elevated TIMP3 may contribute to AD by increasing Aβ production via the APP pathway [38], it might exert a much more complex role in AD, which has to be further clarified.

## 4. Pathway Model

**Figure 3:**
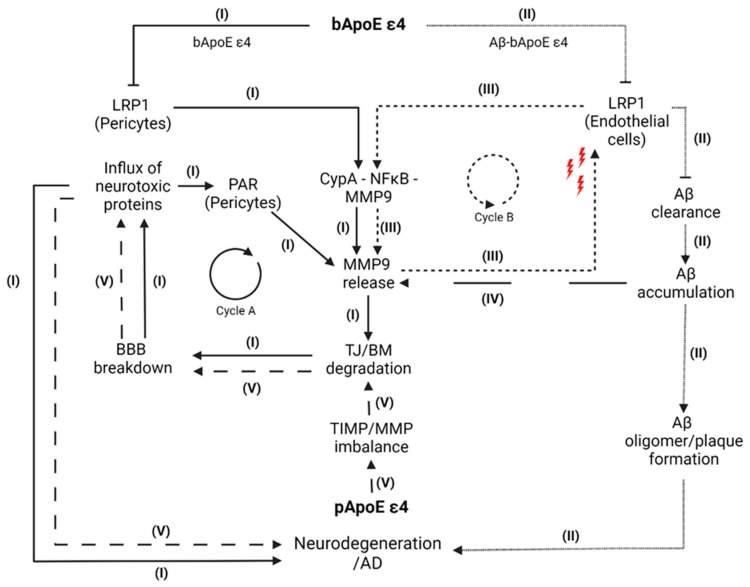
**Pathway model. Path I:** bApoE ε4 cannot prevent the activation of the CypA–NFκB–MMP9 pathway in pericytes resulting in elevated MMP9 levels. Subsequently, MMP9 impairs the maintenance of BM and TJ leading to a BBB breakdown and influx of neurotoxic proteins such as thrombin and fibrinogen, which promote neurodegenerative processes in the brain. Additionally, the elevated thrombin influx might further accelerate BBB disruption via binding to pericytic PARs leading to an MMP9 releasing dysfunctional cycle (Cycle A). **Path II:** In endothelial cells, Aβ–ApoE ε4 complexes redirect the clearance of Aβ from LRP1 to slower VLDLR, which leads to an increase in Aβ molecules resulting in the formation of toxic Aβ oligomers and plaques, which finally promote inflammatory and neurodegenerative processes related to AD. **Path III:** Pericyte-driven release of MMP9 might promote MMP9-driven shedding of LRP1 in endothelial cells leading to an impaired Aβ clearance and increased Aβ levels. The loss of LRP1 might initiate the activation of the CypA–NFκB–MMP9 in endothelial cells leading to an additional MMP9 release, which, in turn, promotes the further shedding of LRP1 (Cycle B). **Path IV:** Paths I and III might be accelerated via the Path II-driven extracellular accumulation of Aβ, which might initiate MMP9 release in endothelial cells leading to BM/TJ degradation (Path I) as well as LRP1 shedding (Path III) resulting in neurodegenerative processes. **Path V:** pApoE ε4 may influence blood-plasma-based factors, which contribute to inflammatory responses in endothelial cells. These contribute to a TIMP/MMP9 imbalance resulting in a BM/ECM dysregulation as well as degradation of TJs. The resulting BBB breakdown enables an influx of a wide range of neurotoxic proteins, which promote AD-related neurodegenerative processes. **Abbreviations:** Aβ = amyloid beta; BBB = blood–brain barrier; BM = basement membrane; bApoE = brain ApoE; CypA = cyclophilin A; ECM = extracellular matrix; LRP1 = low-density lipoprotein receptor-related protein 1; MMP9 = metalloproteinase 9; NFκB = nuclear factor-κB; pApoE = peripheral ApoE; PAR = protease-activated receptor; TIMP = tissue inhibitors of metalloproteinases; TJ = tight junction; VLDLR = very low density lipoprotein receptor. Modified from Zlokovic [7]. Created with BioRender.com accessed on 1 September 2023.

Building upon the above-mentioned findings, we now present a pathway model integrating the three mechanisms by which ApoE might impact BBB stability. An illustration of the model is presented in Figure 3.

To begin with, astrocytic-expressed bApoE ε4 is ineffective at suppressing the pro-inflammatory CypA–NFκB–MMP9 pathway in pericytes (Figure 3, Path I). More specifically, the inability of bApoE ε4 to bind to LRP1 in pericytes cannot prevent the activation of CypA. Consequently, activated CypA leads to NFκB-mediated MMP9 release, which impairs the maintenance of TJs and the BM and results in BBB breakdown [7,11]. Thereby, the resulting influx of blood-derived neurotoxic proteins (e.g., fibrinogen, thrombin, albumin) accelerates inflammatory and neurodegenerative processes in the brain, which might contribute to the pathogenesis of AD [33,40]. While fibrinogen is considered to particularly stimulate microglial activation [40], elevated thrombin levels might further promote BBB disruption via binding to pericytic PARs and increasing MMP9 release [33]. Hence, it is conceivable that the influx of thrombin ultimately promotes the initiation of a self-perpetuating dysfunctional cycle [33], which persistently diminishes the BBB in the long term (Cycle A). Thereby, the pericyte-driven release of MMP9 might represent the start of a second dysfunctional cycle (Cycle B). As MMP9 is able to proteolyse LRP1 in endothelial cells [31], it can be assumed that both BBB impairment due to deficient CypA inhibition in pericytes and the resulting influx of thrombin [33] promote MMP9-driven shedding of LRP1 in endothelial cells, which finally leads to impaired Aβ clearance and increased Aβ levels (Path III) [29,31]. Thereby, a loss of LRP1 might initiate the activation of the CypA–NFκB–MMP9 pathway in the endothelium leading to an additional MMP9 release [31]. Given the already weak interaction of Aβ–bApoE ε4 compounds with endothelial LRP1 (Path II), the additional LRP1 loss and elevated MMP9 release (Cycle B) further increase Aβ accumulation contributing to the formation of toxic Aβ oligomers and plaques [29,31], which finally promote inflammatory and neurodegenerative processes related to AD [3,7,27,28]. These processes might be accelerated via the Path II-driven extracellular accumulation of Aβ, which might also initiate MMP9 release in endothelial cells (Path IV) [29].

Furthermore, the presence of pApoE ε4 from the liver may influence blood plasma-based factors, which contribute to inflammatory responses in both endothelial cells and cells of the gliovascular unit leading to BBB breakdown (Path V). This modulation additionally contributes to the degradation of TJs and a dysregulation of the BM and extracellular matrix due to TIMP/MMP9 imbalance as well as increased vessel-associated gliosis [23].

In summary, our proposed model indicates that the described pathways might directly or indirectly contribute to inflammatory and neurodegenerative processes promoting the pathogenesis of AD. They operate predominantly via the development and maintenance of a persistent MMP9 release through Cycles A and B via bApoE ε4 as well as the peripheral modulation of plasma factors via pApoE ε4 [23].

However, this model has to be seen in light of some limitations. Since the underlying biological processes have mainly been studied in cell and mouse models, we cannot make any assertions as to whether the described mechanisms by which ApoE ε4 may impact BBB integrity are transferable to humans. Also, as we were not able to test our model with mechanistic wet lab experiments, additional studies are needed for verification. Moreover, this model focuses on the role of MMP9 and represents only an excerpt of the processes affecting BBB stability. For instance, LRP1 [41] and TIMP3 [36] exert much more wide-ranging physiological roles and might be involved in additional pathophysiological processes. Therefore, further studies are warranted to bridge this gap. Furthermore, since this is not a systematic review, we did not perform a structured, systematic literature search. Instead, we have concentrated on the most recent and robust studies regarding the influence of ApoE ε4 on BBB integrity, which have offered a deeper understanding of the current state of knowledge. We have made every effort to include all available evidence, but we cannot ensure absolute comprehensiveness.

## 5. Future Perspectives

As visualised by our supposed model, both bApoE ε4 and pApoE ε4 are likely involved in neurodegenerative processes via BBB integrity impairment or Aβ accumulation, contributing to the pathogenesis of AD. Disrupting the balance of the BBB leads to neuronal damage and impedes Aβ clearance at the gliovascular unit, potentially creating a harmful neurodegenerative environment via a persistent MMP9 release. Although cell and mouse models suggest that AD-related pathologies have a significant impact on each component of the gliovascular unit, additional clinical studies are needed to determine whether BBB disruption is an essential characteristic of AD development and progression.

Despite the extensive research in the field, several open questions and scientific challenges persist. These include a more detailed investigation of blood-derived proteins regarding their neurotoxicity and their specific roles in the pathogenesis of AD. In addition to thrombin [33,42], it is assumed that fibrinogen isoforms exert a significant role in the vascular pathology of AD and neuroinflammation [43]. With regard to BBB breakdown, fibrinogen induces a pathogenic response in microglia, contributing to oxidative stress, inflammation and neurodegeneration [35], leading to dendrite loss as well as dendritic spine elimination and cognitive decline in an AD-related manner [40]. This is underpinned by the observation that the removal of fibrinogen can largely reverse such blood-induced microglia neurodegenerative signatures [35].

In addition, it remains elusive whether an ApoE ε4-driven neurotoxic protein influx promotes neurodegenerative processes in general or if it is restricted to AD-specific brain areas. According to previous research, the initial manifestation of AD pathology occurs in the transentorhinal cortex and progressively extends into the entorhinal cortex and hippocampus, two integral parts of the limbic system [44]. Cross-sectional and longitudinal analyses of regional grey matter volumes and grey matter atrophies showed that hippocampal and entorhinal cortex volumes were the most vulnerable areas in human non-demented ApoE ε4 carriers [45,46]. Research findings also indicate that ApoE ε4 mice show restricted neuronal dendritic arborization in the hippocampus, characterised by reduced branching and spine density, as well as a decrease in their complexity within the entorhinal cortex [47]. Additionally, the examination of BBB permeability revealed heightened BBB disruption in the hippocampus and parahippocampal gyrus among cognitively healthy individuals carrying the ApoE ε4 allele (ε3/ε4 and ε4/ε4) in comparison with cognitively healthy individuals with the ApoE ε3 homozygous genotype (ε3/ε3) [13,14]. Although these results suggest a brain area-specific effect of ApoE ε4 on BBB integrity, more research is needed to confirm these observations.

Another open question is how such an ApoE ε4-driven BBB breakdown can be avoided. Possibly, the inhibition of MMP9 release might be a promising target to prevent TJ and BM degradation as well as LRP1 shedding. For instance, treatment of ApoE ε4 mice with the immunosuppressive intracellular CypA-binding drug cyclosporine A indicated that BBB changes are reversible and cyclosporine A might be a promising drug approach to prevent bApoE ε4-induced BBB breakdown [11]. A similar effect was reported for the non-immunosuppressive CypA inhibitor Debio-25, which was shown to protect against neurodegeneration even in the presence of LRP1 endothelial deficiency [31]. Besides CypA inhibitors, NFκB inhibitors such as pyrrolidine dithiocarbamate (PDTC) might be interesting targets to prevent BBB breakdown. Experiments indicate that PDTC can reduce MMP9 activation and reverse BBB breakdown in mice [11]. Additionally, Shackleton and colleagues showed that the treatment of human brain microvascular endothelial cells and ApoE ε4 mice with the MMP9 inhibitor SB-3CT can significantly improve Aβ removal through the prevention of LRP1 shedding [29]. Although these drugs targeting the CypA–NFκB–MMP9 pathway represent promising treatment approaches, additional studies are needed to verify their efficacy related to AD in clinical studies.

Regarding pApoE, Liu and colleagues showed that the addition of exogenous TIMP3 led to enhanced TJs in the presence of pApoE ε4 [23]. Hence, TIMP3 might play a key role in BBB maintenance via MMP inhibition. It is conceivable that a TIMP3 replenishment or plasma exchange in ApoE ε4 carriers contributes to the maintenance of BBB integrity by affecting the TIMP3-mediated pathway or restoring the TIMP3/MMP9 balance, respectively. TIMP3 might, therefore, represent an interesting drug target in preventing TJ and BM disruption and protecting BBB integrity. However, as reports on the application of TIMP3 in neurodegenerative diseases are limited, so far, treatment approaches remain elusive and should be further explored.

In addition to BBB-stabilising medications, the selective opening of the BBB to systematically administer therapeutic drugs into the parenchyma of neural tissues has been proposed as another interesting treatment approach [22]. However, considering the impact of ApoE ε4 on BBB integrity, such approaches should be handled with care, especially for ApoE ε4 carriers. It is essential to ensure that unintended neurotoxin influx is prevented and that the BBB can be restored. For instance, increased BBB permeability is implicated in postoperative neurocognitive impairments following peripheral surgery with inhalation anaesthetics such as sevoflurane or isoflurane [48]. Similar findings were reported in animal research, where particularly older sevoflurane-treated rats showed increased BBB permeability and failed to regain BBB integrity within 24 h of exposure to anaesthetics [49]. Consequently, since BBB integrity in older ApoE ε4 carriers might already be weakened due to a bApoE ε4-driven elevated MMP9 release as well as pApoE ε4-driven TIMP/MMP imbalance, drug approaches that result in targeted or unintended alteration of BBB permeability might increase the risk of advanced neurodegenerative processes.

## 6. Conclusions

This work presents an overview of how brain-derived and peripheral ApoE ε4 might be involved in blood–brain barrier breakdown and promote neurodegenerative processes possibly leading to Alzheimer’s disease. To the best of our knowledge, this is the first model that outlines the impact of both bApoE ε4 and pApoE ε4 on BBB integrity, focusing on detrimental neurodegenerative cycles triggered by MMP9 release and LRP1 shedding. Hence, our model can provide valuable insights for researchers studying neurodegeneration and the role of ApoE ε4 in Alzheimer’s disease. However, future work is needed to verify the herein outlined impact of bApoE ε4 and pApoE ε4 on the pathogenesis of AD. Lastly, further research is warranted to unravel AD-specific effects of neurotoxic proteins, their potential interactions with ApoE ε4/Aβ, the possible wide-ranging physiological and pathological roles of LRP1 and TIMP3 in AD pathogenesis as well as the development of potential treatment approaches to prevent or repair ApoE ε4-related BBB impairments.

## Data Availability

Not applicable.
